# Qualitative exploration of why people repeatedly attend emergency departments for alcohol-related reasons

**DOI:** 10.1186/s12913-017-2091-9

**Published:** 2017-02-16

**Authors:** Tom Parkman, Joanne Neale, Ed Day, Colin Drummond

**Affiliations:** 1 National Addiction Centre, Institute of Psychiatry, Psychology and Neuroscience (IOPPN), King’s College London, Addictions Sciences Building, 4 Windsor Walk, Denmark Hill, London, SE5 8BB UK; 2 Centre for Social Research in Health, University of New South Wales, Sydney, NSW 2052 Australia

**Keywords:** Alcohol, Frequent attender, Emergency departments, Andersen’s behavioural model, Qualitative research

## Abstract

**Background:**

Understanding why people repeatedly attend Emergency Departments (EDs) for alcohol-related reasons is an important prerequisite to identifying ways of reducing any unnecessary demands on hospital resources. We use Andersen’s Behavioural Model of Health Services Use to explore factors that contributed to repeat ED attendances.

**Methods:**

Qualitative interviews were conducted with 30 people who repeatedly attended EDs for alcohol-related reasons (≥10 attendances in the past 12 months). We recruited participants from 6 EDs in London, United Kingdom. Data on socio-demographic characteristics, substance use, contact with specialist addiction and other health services, most recent ED attendance, and previous ED attendances were analysed.

**Results:**

Participants reported long-standing health problems, almost all were unemployed, and many had limited education and unstable housing. Most held positive health beliefs about EDs, despite some negative experiences. They reported limited community resources: poor social support, inaccessible primary care services, dislike or lack of information about specialist addiction services, and difficulties travelling to services. In contrast, EDs offered immediate, sympathetic care and free transport by ambulance. Participants’ perceived need for care was high, with physical injury and pain being the main reasons for ED attendance.

**Conclusions:**

Push’ and ‘pull’ factors contributed to repeated ED use. ‘Push’ factors included individual-level problems and wider community service failings. ‘Pull’ factors included positive experiences of, and beliefs about, ED care. Community services need to better engage and support people with complex drinking problems, whilst ED staff can be more effective in referring patients to community-based services.

## Background

People who repeatedly attend Emergency Departments (EDs) for alcohol-related reasons comprise a relatively small patient population but account for a disproportionate use of ED resources [1]. Their hospital attendances place strain on healthcare infrastructures and are costly for national public healthcare systems [2–5]. This has generated concern in many different countries [6–16]. In the UK, for example, the pressure placed on the National Health Service (NHS) by people with alcohol-related problems who attend EDs unnecessarily has been widely documented [17–21].

To-date, the international literature on people who repeatedly attend EDs because of alcohol has been largely descriptive and derived from quantitative surveys or epidemiological studies exploring prevalence and socio-demographic characteristics, such as gender, age, employment history, income, and education. Studies indicate that members of this patient population tend to be male [11] and over 45 years of age [8]. They are also likely to have personal histories of homelessness and other substance use problems, be heavy smokers [8, 11], have limited education, experience high levels of mental health problems, and live in socio-economically deprived areas [7, 12, 15, 22].

The reasons why people repeatedly attend EDs for alcohol-related reasons cannot easily be determined from the available quantitative survey and epidemiological data and thus remain unclear. Potential explanations can, however, be explored using a qualitative study design, supported by a theoretical framework. Andersen’s Behavioural Model (BM) of Health Services Use [23, 24] is an established conceptual model designed to understand and explain why people use health care services [25]. It incorporates individual, community and wider health system determinants [25, 26], so facilitating the analysis of both personal and contextual factors relating to health service utilisation.

The BM was developed nearly 50 years ago but is still widely used [26–28]. Although it has evolved over time, it still has, at its core, three sets of predictive factors: ‘*predisposing’*, ‘*enabling*’ and ‘*need*’ [24]. ‘Predisposing factors’ refer to personal characteristics that predate ill-health but affect an individual’s propensity to use health services. They include demographic characteristics (gender, age, ethnicity, previous health problems, and relationship status); social structure (an individual’s education, housing, and occupation); and health beliefs (the positive and negative attitudes people have about healthcare services).

The inclusion of ‘enabling factors’ in the BM is in recognition of the fact that whilst individuals may have predispositions to use health care, their actual usage will depend on them being able to access services. Enabling factors exist at the individual and community level and include income, whether or not an individual has a regular source of care and the nature of that care, the availability of services, information about services, and an individual’s resources or means to access services. ‘Need factors’ refer to an individual’s own assessment of their health status and requirement for medical care (‘perceived’ need), as well as their health status and requirement for care as judged by professionals (‘evaluated’ need). Examples of need factors include number, strength and duration of symptoms and restrictions on activities of daily living.

The aim of this paper is to understand why people repeatedly attend EDs for alcohol-related reasons in order to identify how we might reduce any unnecessary demands on hospital resources. To this end, we use Andersen’s Behavioural Model of Health Services Use to identify predisposing factors, enabling resources and needs that contribute to repeated ED attendances.

## Methods

### Study design and setting

Between March 2015 and June 2105, we conducted semi-structured face-to-face qualitative interviews with 30 people who repeatedly attended EDs for alcohol-related reasons. A sample size of 30 was chosen on the grounds that this was practical and achievable within the study time frame and would generate sufficient data for the identification of themes, concepts and categories. Participants were identified from EDs in six hospitals located in socio-economically diverse areas of south and west London, United Kingdom (UK). As we anticipated difficulties recruiting participants and organising the interviews, we identified EDs situated within a 2-h travel time from our place of work. This enabled us to maximise recruitment whilst minimising research costs. The study received ethical approval from a UK NHS research ethics committee (REC reference number: 14/LO/1251).

### Selection of participants

Inclusion criteria for the study were *“any patient aged 16 or over who attends any Accident and Emergency department 10 or more times within a year or 5 or more times within a 3*-*month period for an alcohol*-*related condition”* [29]. ED nurses, alcohol liaison nurses, and specialist alcohol staff working in the six hospitals reviewed their patient records to identify potential participants meeting the above study inclusion criteria. The same staff then approached these potential participants, explained the study to them, and provided details on what participation would involve. If a patient expressed interest, the staff member gained verbal consent for their contact details to be passed on to the study’s researcher (TP). TP then contacted interested patients by telephone, explained the study again and arranged a time, date and place to conduct the interview. Patients were contacted until 30 interviews had been completed. Only three individuals could not be contacted by TP and only one declined an interview.

### Data collection

Interviews were conducted by TP during the day, typically in the morning so that participants had less time to drink before the interview. Prior to each interview, TP provided written and further verbal explanation about the study and secured written informed consent. If individuals could not read or write (*n* = 2), TP read out the information sheet, asked if they had any questions, and helped them to complete the consent form. Most of the participants (*n* = 23) wanted to be interviewed in their home. When individuals did not have stable housing or expressed a desire to be interviewed elsewhere, a suitable alternative location (hostel, doctor’s surgery, restaurant, or nursing home) was used.

Although some participants explained that they had drunk before the interview or drank during the interview, TP judged all of them to be sober enough to knowingly consent and participate in the research. Each interview lasted between 60 and 120 min, was guided by a semi-structured topic guide, and was audio recorded. In addition to participants’ socio-demographic characteristics and past and present alcohol, tobacco and drug use, the topic guide included open-ended questions on their contact with specialist addiction services, contact with wider health and social services, most recent ED attendance, previous ED attendances, and types of support (if any) desired for ongoing alcohol use.

At the end of the interview, TP helped all participants to complete the Severity of Alcohol Dependence Questionnaire (SADQ) [30]. This is a 20-item measure of the severity of alcohol dependence, which scores from 0–60. A score of less than 16 indicates ‘mild alcohol dependence’, 16–30 indicates ‘moderate alcohol dependence’, and 31–60 indicates ‘severe alcohol dependence’. Hospital staff also provided information on each individual’s number of ED attendances and hospital admissions from ED in the last 12 months. On completion of their interview, all participants were given a £15 voucher in recognition of their time.

### Analysis

Audio recordings were transcribed verbatim by a professional transcription service and entered into the qualitative software program MAXQDA™ (version 10) for systematic coding. A coding framework based on deductive codes derived from the topic guide and inductive codes that emerged from topics discussed spontaneously during the interviews was developed. Each interview transcription was then reviewed line-by-line with all the interview data indexed to one or more relevant codes. To address the aim of this paper, all inductive and deductive codes relating to participants’ socio-demographic characteristics, alcohol and other drug use, contact with specialist addiction and other health services, most recent ED attendance, and previous ED attendances were analysed.

First, each code was exported from the specialist qualitative program into its own Microsoft Word document and reviewed line by line, using a systematic and transparent process known as Iterative Categorisation [31]. This generated emergent themes that were then mapped, as closely as possible, onto Andersen’s three sets of predictive factors: ‘predisposing factors’, ‘enabling factors’ and ‘need’. The range and nature of the data within each component of Andersen’s model were then reviewed and reported, with quotations used to illustrate key findings. In addition, mean scores for the SADQ, ED attendances, and hospital admissions were calculated using SPSS version 23™ [32]. All of the analyses were conducted by TP and JN. To protect their anonymity, participants were given pseudonyms.

## Results

### Predisposing factors

#### Demographic characteristics

Table 1 shows the demographic characteristics of the study participants. They included 18 men and 12 women; their ages ranged from 20 to 68 years (mean = 47.9 years) and they had a range of ethnic backgrounds. Although most (*n* = 26) were single, 2 were married, one was divorced and one was cohabiting.

**Table 1:** Demographic characteristics

Characteristic	Participant
Gender	
Male	18
Female	12
Age (years)	
Mean (range)	47.9 (20–68)
Ethnicity	
White British	19
Asian British	4
Mixed race (British)	3
European	3
Other	1
Physical illness	
Currently experiencing physical illness	26
Physical illness reported for many years	25
Physical illness for less than 1 year	1
Reported good health	4
Relationship status	
Single	26
Married	2
Divorced	1
Cohabitating	1

Nearly all participants reported chronic physical health problems (including, poor kidney functioning or kidney failure, diabetes, gastritis, hepatitis, pancreatitis, high blood pressure, angina, respiratory problems, incontinence, and peripheral neuropathy). Many also referred to acute health problems (such as strokes and heart attacks, and pain from injuries sustained as a result of drinking-related accidents), as well as general life-style related health problems (such as tooth decay, sleeping difficulties, and malnutrition). All participants complained of poor mental health and nearly a third said that they had received a formal mental health diagnosis (such as depression, bipolar disorder, bulimia, vascular dementia, borderline personality disorder, and emotionally unstable personality disorder).

Most participants also recognised that they had a drink problem, referring to withdrawals, an inability to stop drinking, and increasing tolerance:

“*I drink anything I can get my hands on really… I have even drunk toilet bleach a couple of times when they* [the local supermarket] *wouldn*’*t give me any.”* (Philippa; 52 years)

The types of drinks participants consumed included beer, cider, wine and spirits, with most reporting that they drank a combination of beverages (mostly, beer and/or cider and spirits). Those who only drank beer or cider typically consumed 10–15 cans a day (usually 7–9% alcohol by volume [ABV]). Other participants typically drank fewer cans of beer or cider (usually 5–8 cans) and between half and a bottle of spirits. A minority of participants only drank spirits, some mixing these with non-alcoholic drinks and others drinking them neat. At the time of interview, over two-thirds said that they smoked, but only one discussed current illicit drug use (reference removed for review).

#### Social structure

Most of our participants had relatively low levels of formal education (see Table 2).

**Table 2:** Social structure factors

Characteristic	Participant
Education	
None	9
GCSE (usually taken at age 16 years)	12
>5 GCSEs	12
A-level	5
University degree	3
PhD	1
Housing	
Local authority/housing association	8
Street homeless	6
Hostel/YMCA/sheltered housing	5
Owner occupied	4
Private renting	3
Living with friends/family	3
Medical Centre	1
Employment	
Unemployed	28
Employed	2
Prison	
No	24
Yes	6

Nearly a third reported no school education and just over a third had left school at 16 years of age with five or less qualifications. Four, however, had a university level qualification. Many participants had relatively unstable housing circumstances – living on the streets or in temporary accommodation such as hostels. Despite this, nearly a third lived in social housing and small numbers rented, lived with family or friends, or owned their own home. Almost all were unemployed and only a minority had ever been in prison:

“*I got nothing really*… *No house*… *never worked*… *living here* [a hostel]… *and my benefits aren*’*t coming in properly neither.”* (Jack; 53 years)

#### Health beliefs about EDs

Participants mostly gave positive accounts of their past ED attendances, “*I*’*d say 99*% *of them* [attendances] *were very good”* (Luke; 56 years). Specifically, they referred to being treated ‘nicely’, ‘fairly’, ‘respectfully’, ‘politely’, ‘empathetically’, ‘compassionately’, and ‘with honesty’ by ED nurses, doctors and paramedics:

“*She* [doctor] *was abrupt*, *but she did try and help me*… *I liked that she was honest.”* (Emma; 47 years)

In addition, most participants described EDs as relatively attractive places, stating that they were warm, safe, always open, and provided immediate medical help, often with a free cup of tea and a sandwich. Several participants expressed appreciation at the fact that ED staff had sometimes washed their soiled clothes or occasionally telephoned them post discharge to check how they were. Some also believed that ED staff provided good care because they knew their personal histories:

“*I like them. They treat me well*… *and they know what*’*s my problem. So they know how to deal with me better*.” (Polly; 20 years)

Although some participants described negative ED experiences, these were comparatively rare. Specifically, participants reported instances where they felt that they had not been taken seriously, were ignored, treated rudely by nurses, verbally abused by doctors, or did not receive the treatment they felt they needed. For example, one man said that a nurse had been talking on her mobile phone whilst giving him an intravenous line. Several participants also complained that they had had their medication taken away without explanation by ED staff and one man described being physically assaulted by a paramedic:

“*There was one ambulance man*… *I was trying to say I was having a panic attack. He thought I was trying to put on a seizure*, *fake it. He called it a ‘pseudo*-*seizure*’… *I was just panicking. And so he took a nasal cannula and shoved it down my nose and said*, ‘*if you*’*re going to fuck about with me*, *we*’*re going to fuck about with you*.” (Nick; 24 years)

Participants explained that these negative experiences had left them feeling depressed, angry and frustrated. Nonetheless, they had all returned to EDs subsequently:

“*I just prefer it* [ED]… *I get treated quick*… *and they*’*re* [staff] *nice to me*.” (Wayne; 58 years)

### Enabling resources

#### Individual resources

Overwhelmingly, our participants had low incomes. Almost all were in receipt of state benefits and only two were in paid work. In terms of personal care and support, our participants mostly reported that they had either no or only negative relationships with family members and few friends or social contacts. Indeed, many stated that they only socialised with other heavy drinkers. Although a minority retained some positive relationships, which they believed protected them from drinking, most said that they had little social support:

“*I don*’*t have that kind of circle of friends*, *people to rely on*, *you know*.” (Jack; 53 years)

#### Community resources

Many participants explained that they tended to go to EDs at night or at weekends when their doctors’ surgeries were closed. Others explained that they would try to wait for appointments with their general practitioner but long waiting times (sometimes weeks) meant that medical problems were not resolved quickly enough so they attended an ED instead. Further, all participants reported that they routinely called ambulances as this provided a quick and easy route to care:

“*I am a sick man. I need help*… *I always call an ambulance because I cannot get there*.” (Eric; 49 years)

At the time they were interviewed, less than a third of our participants said they were receiving support from a specialist addiction service. When those not accessing specialist support for their drinking were asked why, reasons were varied. A few reported that they did not need help from a specialist service, a few said they did not know what services or types of support were available, a few said that they had previously found specialist addiction services unhelpful or judgemental, and a few said that their health problems, particularly walking problems, made it difficult for them to get to services:

“*I don*’*t know how many times I*’*ve called* [an ambulance]… *I have to*… *I can*’*t walk anywhere really*.” (Michelle; 25 years)

Most participants stated that they had never received any information from ED staff about community services that could help them with their drinking. Similarly, most said that they had never been referred to a specialist community addiction service by ED staff. Meanwhile, those participants who had been referred commented that this had not been helpful as it was invariably back to a service that they had previously used but disliked:

“*They send me back to* [community addiction service]. *I*’*m not going because they*’*re not helping*, *actually* [they’re] *killing* [me]. *It*’*s just rabbit*, *rabbit*, *rabbit* [talking]. *It*’*s no good*.” (Deborah; 36 years)

### Need

#### Perceived need

When participants were asked why they kept attending ED, their most common explanation was that they needed immediate medical attention for physical injuries and associated pain. Further, they identified alcohol as the underlying cause of most of these injuries:

“*Bruised my leg*, *had a broken arm*, *broken fingers*, *broken jaw*… [I] *get so pissed I*… *just fall over*.” [Simon; 42 years]

Other commonly reported reasons for ED attendance were also alcohol-related, and included general intoxication, poor health after excessive periods of drinking, and severe alcohol withdrawal symptoms (seizures, shaking, sweating, and convulsions). Sometimes participants reported that they attended ED because they could not obtain alcohol and were afraid of imminent withdrawal onset. These individuals invariably explained that they went to ED for medication to prevent and ease the anticipated withdrawal symptoms:

“*Rather than face withdrawals*… *I call an ambulance*… *I*’*d rather kill myself than face it* [withdrawal].” (Jack; 53 years)

A small number of participants stated that they were so anxious about going into withdrawal that they called ambulances using false reasons. For example, Matthew said that he would claim that he felt suicidal or would threaten to attempt suicide as he knew that the ambulance would then come more quickly:

“*Sometimes I have to wait hours* [for an ambulance]… *so I might make something up*… *like I feel suicidal*… *I*’*d never do it* [attempt suicide], *but they always come quickly then*… *Seems the only thing they take seriously*.” (Matthew; 50 years)

Additionally, our participants commented that they often attended EDs because of pain and complications associated with longstanding physical health conditions unrelated to their alcohol use. Equally, some said they went because of health problems or physical injuries caused by sleeping on the streets, poor housing, or domestic violence:

“*I*’*ve been beaten many times. That*’*s why I been to* [ED] *so many times*.” (Emma; 47 years)

Lastly, some participants highlighted how they attended EDs because of injuries caused by self-harming, suicide attempts and deliberate overdosing. For example, several described cutting themselves, drinking highly toxic substances (such as bleach or paint remover), or taking large quantities of pills (often combined with drinking). As one man diagnosed with bulimia, agoraphobia, anxiety and depression commented:

“*When I*’*m alone*, *the thoughts are destructive*, *very destructive*… *I got really drunk one night*… *and I stabbed myself in the legs eighteen times and left myself in a wheelchair for four months*… *I wanted to end it* [life].” (Nick; 24 years)

#### Evaluated need

Our data collection did not include professionals’ views of our participants’ health needs, but we are able to report more objective information on their health status via the SADQ and their hospital records data. Our participants’ mean total SADQ score was 32.53 (SD = 13.98; range = 2–55) indicating ‘severe alcohol dependence’. In total, 17 participants scored as ‘severely alcohol dependent’, 10 as ‘moderately alcohol dependent’, and only 3 as ‘mildly alcohol dependent’. Hospital record data indicated that during the past 12 months, our participants had attended ED a mean of 24 times (range = 10–84), with a mean of 5 (range = 0–17) hospital inpatient admissions from ED.

## Discussion

Our study sought to provide insights into the reasons why people repeatedly attend EDs for alcohol-related reasons. This information is needed in order to identify how we might reduce any unnecessary demands on hospital resources. Analyses of the accounts of people who repeatedly attended EDs for alcohol-related reasons were fitted to Andersen’s BM of Health Service Use and this revealed a range of predisposing, resource and need-related factors, which operated at an individual, community and wider health system level.

In terms of predisposing factors, many of our participants reported personal characteristics that increased their likelihood of using health services. In particular, they described high levels of chronic and acute physical and mental health problems as well as problem drinking. Health problems were likely to have been exacerbated by poor housing, homelessness and unemployment. The BM posits that people’s demographic and social situation may be related to their use of healthcare services [23, 24, 33], but does not explain which healthcare services are more likely to be utilised. Our participants described some negative experiences within EDs that were consistent with healthcare providers’ stigmatizing attitudes and behaviour reported elsewhere [22, 34, 35]. Nonetheless, most spoke favourably of their ED visits and this seemed to translate into positive health beliefs about EDs that encouraged repeat attendances.

In the UK, ED care is free at the point of use. Low income would not therefore have prevented our participants from attending EDs, although it would have made it difficult for them to have accessed private health care or private residential drug and alcohol treatment facilities. Compounding their low income, most participants indicated that they had limited social support to help them with their drinking or wider heath needs. They also deemed primary health care services to be inaccessible due to closure at weekends and evenings and long waiting times. Further, participants said that they did not attend specialist addiction services because of lack of information regarding the support available, dislike of specialist services, and mobility problems that made it difficult for them to get to services. In contrast, ambulances provided free and rapid access to health care, again increasing the attractiveness of the ED.

The perceived health needs of our study population were high. Reflecting this, they reported that they attended EDs for physical injuries and pain. These were often, but not always, alcohol-related; for example, pain was sometimes caused by chronic health problems and injuries related to difficult housing and social circumstances and poor mental health. Our participants also attended EDs because of actual or impending alcohol withdrawal symptoms, a phenomenon that can be life-threating [36]. SADQ scores confirmed that over two thirds of our participants were ‘severely alcohol dependent’. In addition, the mean numbers of admissions to hospital wards from ED within the last 12 months was 5 (range 0–17), indicating that hospital staff also evaluated their health needs as sufficient to warrant inpatient care.

Borrowing terminology from the field of human geography where a ‘push’ factor is a force that drives an individual to leave a place and a ‘pull’ factor is what draws them to a location [37], our analyses suggest that both ‘push’ and ‘pull’ factors contributed to repeat ED attendances amongst this drinking population. ‘Push’ factors included individual problems (such as poor physical and mental health, low income, limited or no social support), and wider community and health system problems (such poor and/or slow access to primary health care and specialist addiction services and limited information on available services). ‘Pull’ factors included positive experiences of, and beliefs about, EDs (they are warm, safe and always open) and ED staff (they understand individual patient needs and treat them respectfully and sympathetically). Furthermore, ambulances provided a quick and easy route to hospital that did not incur a personal financial cost.

From our findings, it at first seems difficult to identify interventions or strategies that might be introduced into ED settings to reduce the demand on hospital resources by people with complex alcohol problems. Advocating slower ambulance responses, less sympathetic treatment by ED staff, or making patients with alcohol problems wait before receiving ED care are unacceptable and unethical when patients report high physical and mental health care needs, including symptoms that may be life-threatening. The more obvious approach, although not to our knowledge previously reported in the literature as a potential solution to repeated ED attendance for alcohol-related problems, would be to find ways of making primary health care and specialist addiction services more and appealing to, and appropriate for, this population. To this end, our findings highlight the need for more out-of-hours support, a fast-track into community services, and personalised assistance with a wide range of health, housing and social problems, including travelling to services. Services would also likely need to advertise themselves more widely and treat problem drinkers more sensitively.

Nonetheless, we maintain that ED staff could play a greater role in preventing at least some unnecessary ED attendances. Repeated ED use by a patient population that is otherwise very difficult to engage indicates that those with complex drink problems routinely trust and value ED staff. Most participants in our study reported that they had never received any information about, or been referred to, specialist community addiction services by ED staff, or when they had been referred this was simply back to services they disliked. This seems like a missed opportunity. With additional training on alcohol dependence, up-to-date information on the various specialist services that can help people with drink problems, and closer relationships with named sympathetic workers based in local alcohol and other primary care services, ED staff might be able to signpost and refer patients more effectively. Through careful liaison with, and personal recommendations of, non-emergency services, ED staff could potentially extend the trust patients have in them to those working in community settings.

### Study limitations

Our analyses are exploratory and based on a relatively small number of qualitative interviews conducted with individuals recruited from only six hospitals across London, UK. It is inappropriate to draw empirical generalizations from our data and caution should be taken when considering our findings in relation to other locations where health care systems may be different. We relied on participants’ self-report data and did not attempt any independent verification of their accounts. Equally, we did not have access to professionals’ views of our participants’ health needs when considering evaluated need; instead we relied on a single validated alcohol scale and hospital records data.

## Conclusions

Andersen’s BM of Health Service Use has provided a useful framework for exploring the reasons why people repeatedly attend ED for alcohol-related reasons. The model points to a population with high physical and mental health care needs that often require urgent medical attention. It is therefore unsurprising that they find their way to EDs and return there following positive treatment experiences. In contrast, the BM revealed how primary care and specialist addiction services frequently fail to engage, support and meet the complex needs of this group of drinkers [38, 39]. This appears to be a missed opportunity for preventing medical emergencies that could be addressed elsewhere in the healthcare system prior to a medical crisis - particularly those underpinned by alcohol, mental health, long-standing physical health or other wider social problems.

In conclusion, we suggest that the best places to develop and deliver interventions and strategies to reduce repeat attendances to ED for drink-related reasons are within primary care and specialist addiction services rather than within EDs themselves. However, we maintain that ED staff have an important role to play in both treating patients’ acute health problems and referring them to community-based services when these are more appropriate. With careful planning and sensitive liaison, ED staff can potentially extend the trust patient have in them to those working in other settings, thereby better bridging the gap between expensive emergency and less expensive community care. Over time, this should help to reduce at last some unnecessary demands on hospital resources.

## Abbreviations

BM: Behavioural Model
ED: Emergency Department
NHS: National Health Service
SADQ: Severity of Alcohol Dependence Questionnaire
UK: United Kingdom

## References

[1] LaCalle E, Rabin E (2010). Frequent Users of Emergency Departments: The Myths, the Data, and the Policy Implications. Ann Emerg Med.

[2] Thornquist L, Biros M, Olander R, Sterner S (2002). Health care utilization of chronic inebriates. Acad Emerg Med.

[3] Hansagi H, Engdahl B, Romelsjo A (2012). Predictors of Repeated Emergency Department Visits among Persons Treated for Addiction. Eur Addict Res.

[4] Mandelberg JH, Kuhn RE, Kohn MA (2000). Epidemiologic analysis of an urban, public emergency department’s frequent users. Acad Emerg Med.

[5] Shumway M, Boccellari A, O’Brien K, Okin RL (2008). Cost-effectiveness of clinical case management for ED frequent users: results of a randomized trial. Am J Emerg Med.

[6] Brubacher JR, Mabie A, Ngo M, Abu-Laban RB, Buchanan J, Shenton T (2008). Substance-related problems in patients visiting an urban Canadian emergency department. Canadian Journal of Emergency Medicine.

[7] Curran GM, Sullivan G, Williams K, Han XT, Collins K, Keys J (2003). Emergency department use of persons with comorbid psychiatric and substance abuse disorders. Ann Emerg Med.

[8] Fleming EA, Gmel G, Bady P, Yersin B, Givel JC, Brown D (2007). At-risk drinking and drug use among patients seeking care in an emergency department. J Stud Alcohol Drugs.

[9] Hannon MJ, Luke LC (2006). The burden of alcohol misuse on the emergency department. Ir Med J.

[10] Rockett IRH, Putnam SL, Jia HM, Chang CF, Smith GS (2005). Unmet substance abuse treatment need, health services utilization, and cost: A population-based emergency department study. Ann Emerg Med.

[11] Whiteman PJ, Hoffman RS, Goldfrank LR (2000). Alcoholism in the emergency department: An epidemiologic study. Acad Emerg Med.

[12] Moore G, Gerdtz M, Manias E, Hepworth G, Dent A (2007). Socio-demographic and clinical characteristics of re-presentation to an Australian inner-city emergency department: implications for service delivery. BMC Public Health.

[13] Charalambous MP (2002). Alcohol and the accident and emergency department: A current review. Alcohol Alcohol.

[14] Dent A, Hunter G, Webster AP (2010). The impact of frequent attenders on a UK emergency department. Eur J Emerg Med.

[15] Williams ERL, Guthrie E, Mackway-Jones K, James M, Tomenson B, Eastham J (2001). Psychiatric status, somatisation, and health care utilization of frequent attenders at the emergency department - A comparison with routine attenders. J Psychosom Res.

[16] Hansagi H, Olsson M, Sjoberg S, Tomson Y, Goransson S (2001). Frequent use of the hospital emergency department is indicative of high use of other health care services. Ann Emerg Med.

[17] Iacobucci G. Nearly 40% of hospitals missed emergency department waiting time target in last quarter. BMJ. 2013;346:f3618. 10.1136/bmj.f3618 23736801

[18] HM Government (2012). The Government’s Alcohol Strategy.

[19] Iacobucci G. Public health—the frontline cuts begin. BMJ. 2016;352:i272. 10.1136/bmj.i272 26791557

[20] Public Health England. Response to consultation on alcohol indicator published 2013 [cited 8 Apr 2016]. Available from: https://www.gov.uk/government/news/response-to-consultation-on-alcohol-indicator-published.

[21] Department of Health. Health Lives, Health people: Improving Outcomes and Supporting Transparency. A Public Health Outcomes Framework for England, 2013–2016. London: Department of Health; 2012.

[22] Neale J, Parkman TJ, Day E, Drummond C. Sociodemographic characteristics and stereotyping of people who frequently attend Accident and Emergency departments for alcohol-related reasons: Qualitative study. Drugs: Education Prevention and Policy. in press.

[23] Andersen RM (1968). Behavioural Model of Families’ Use of Health Services.

[24] Andersen RM (1995). Revisiting the Behavioral Model and Access to Medical Care: Does It Matter?. J Health Soc Behav.

[25] Petrovic K, Blank TO (2015). The Andersen–Newman Behavioral Model of Health Service Use as a conceptual basis for understanding patient behavior within the patient–physician dyad: The influence of trust on adherence to statins in older people living with HIV and cardiovascular disease. Cogent Psychol.

[26] Babitsch B, Gohl D (2012). Lengerke v. Re-visiting Andersen’s Behavioral Model of Health Services Use: a systematic review of studies from 1998–2011. GMS Psychosocial Medicine.

[27] Insaf TZ, Jurkowski JM, Alomar L (2010). Sociocultural factors infuencing delay in seeking routine health care among Latinas: A community-based participatory study. Ethn Dis.

[28] Surood S, Lai DWL (2010). Impact of Culture on Use of Western Health Services by Older South Asian Canadians. Can J Public Health-Revue Canadienne De Sante Publique.

[29] Information Services Division Scotland (2014). Data Dictionary A-Z.

[30] Stockwell T, Murphy D, Hodgson R (1983). The Severity of Alcohol Dependence Questionnaire: Its Use, Reliability and Validity. Br J Addict.

[31] Neale J (2016). Iterative Categoristaion (IC): A systematic technique for analysing qualitative data. Addiction.

[32] SPSS Inc (2013). SPSS Statistics for Windows, Version 23.

[33] Andersen RM, Newman JF (1973). Societal and individual determinants of medical care utilization in the United States. Milbank Mem Fund Q Health Soc.

[34] van Boekel LC, Brouwers EP, Weeghal J, Garretsen HFL (2013). Stigma amongst health professionals towards patients with substance use disorders and its consequences for healthcare delivery: A systematic review. Drug Alcohol Depend.

[35] Jeffrey R (1979). Normal rubbish: Deviant patients in casualty departments. Sociol Health Illn.

[36] Raistrick D, Heather N, Godfrey C (2006). Review of the effectiveness of treatment for alcohol problems.

[37] Short JR (2014). Human Geography: A Short Introduction.

[38] Drummond C, Oyefoso A, Phillips T, Cheeta S, DeLuca P, Perryman K, et al. Alcohol Needs Assessment Research Project (ANARP): The 2004 National Alcohol Needs Assessment for England. London: Department of Health; 2005.

[39] Passetti F, Jones G, Chawla K, Bolland B, Drummond C (2008). Pilot study of assertive community treatment methods to engage alcohol-dependent individuals. Alcohol Alcohol.

